# Purkinje cells: the forest shapes the trees

**DOI:** 10.1186/1471-2202-16-S1-P52

**Published:** 2015-12-18

**Authors:** Benjamin Torben-Nielsen, Erik De Schutter

**Affiliations:** 1 Computational Neuroscience Unit, Okinawa Institute of Science and Technology, 1919-1 Tancha, Onna-son, Kunigami-gun, Okinawa, 904-0495, Japan

## 

Purkinje cells are at the heart of all theories about the olivo-cerbellar circuit as they provide the only output from the cerebellar cortex. Hallmark is their planar morphology in a plane perpendicular to the parallel fibers. Moreover, individual Purkinje cells have very dense dendritic arborizations, a feature commonly referred to as space-filling. In a population, they neatly align and cover the available volume in both transverse and medio-lateral directions, so-called tiling. These remarkable morphological features are assumed to allow neurons to intercept as many parallel fibers inputs as possible; up to 250.000 in individual cells. But it remains an open question how individual cells develop a mono-planar dendrite and how the alignment emerges in a population. Here we address both questions.

We recently developed a computational framework, NeuroMaC, to investigate how neuronal morphologies are shaped by interactions with the environment in which they are embedded [1]. In this approach, large numbers of virtual morphologies are generated simultaneously by repeatedly extending simulated growth-cones that follow growth rules expressed in terms of environmental interactions. In this work, we hypothesized that the peculiar Purkinje cell morphology emerges from three simple interactions with its environment. First, dendritic branches of one neuron are repelled by each other, a process called self-avoidance. Second, dendritic branches of different neurons repel each other. Third, all branches are to some extent attracted to the pia surface. Initial simulated growth-cones where placed on a seven-by-seven lattice with plausible spacing: somata where 25 and 150 micron apart from each other in, respectively, the medio-lateral and transverse directions. We found that these simple rules yielded highly realistic morphologies exhibiting the hallmark planarity in the transverse direction. The planarity emerged because the repulsion between branches forced the neuron to occupy all available space, which was larger in the transverse than in the medio-lateral direction. We validated the resulting morphologies against exemplar data and found a good statistical match. Moreover, alignment and tiling emerged in the population.

We demonstrated that key features of the notoriously complex Purkinje cell morphology could be achieved by solely local repulsive and attractive interactions between simulated growth-cones. Thus, we may conclude that the forest does shape individual neurons. While alignment did emerge to a certain extent, it is hard to validate because there is currently no data available on Purkinje cell forests. We also speculate on the necessity of a global organization mechanism that consistently aligns Purkinje cells in the transverse direction.
